# PINK1 Loss of Function Selectively Alters the Mitochondrial‐Derived Vesicle Pathway

**DOI:** 10.1096/fba.2024-00200

**Published:** 2025-07-10

**Authors:** Charlotte L. Collier, Colleen Ruedi, Naomi J. Thorne, David A. Tumbarello

**Affiliations:** ^1^ Biological Sciences University of Southampton Southampton UK

**Keywords:** lysosome, membrane trafficking, mitochondria, mitochondrial quality control, Parkinson's, vesicle transport

## Abstract

Cell homeostasis and metabolic control require the efficient function of mitochondria and implementation of quality control pathways following damage. Cells have various discrete pathways of mitochondrial quality control (mitoQC) to maintain the healthy network. PINK1 and Parkin are two key players in mitoQC, most highly associated with the ubiquitin‐dependent capture and degradation of whole mitochondria by autophagy. However, these proteins have alternative roles in repair routes directing locally damaged cargo to the lysosome, such as the mitochondrial‐derived vesicle (MDV) pathway. We aimed to clarify the role of PINK1 and determine how its loss of function impacts mitochondrial dynamics and quality control. Results indicate PINK1 knockout (KO) has little impact on whole mitochondrial turnover in response to damage in SH‐SY5Y cells, whereas both PINK1 and Parkin KO cells have healthy mitochondrial networks with efficient ATP production. However, TOM20 positive outer‐membrane and damage‐induced PDH‐positive inner‐membrane MDVs are elevated in PINK1 KO cells. Although, in contrast to Parkin KO, this is not due to a defect in trafficking to a LAMP1‐positive compartment and may instead indicate increased damage‐induced flux. In comparison, loss of Atg5‐dependent mitophagy has no effect on whole mitochondrial turnover and only results in a limited elevation in inner‐membrane MDVs in response to damage, indicating autophagy‐independent mechanisms of whole mitochondrial turnover and a minor compensatory increase in damage‐induced MDVs. Therefore, these data suggest PINK1 and Parkin are dispensable for whole mitochondrial turnover, but following their perturbation have disparate effects on the MDV pathway.

## Graphical abstract

PINK1 and Parkin are essential for the proper function of the mitochondrial‐derived vesicle (MDV) pathway in response to local mitochondrial damage. However, unlike the loss of Parkin function, PINK1 knockout results in an elevated number of MDVs due to enhanced flux rather than impaired trafficking to the lysosome.

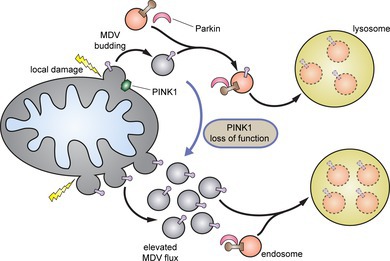

## 1 Introduction

Maintaining cellular homeostasis is a complex process that requires the integration of multiple pathways to limit susceptibility to cell death. The mitochondria lie at the heart of this process due to their essential role in generating ATP by oxidative phosphorylation, while also regulating apoptosis, calcium balance, and organelle lipid transfer [1]. Thus, dysfunctional mitochondria, attributed to aging and diseases such as Parkinson's and cardiovascular disease, will by default lead to an increased propensity for cellular dysfunction. Multiple mitochondrial quality control (mitoQC) mechanisms exist to selectively remove damaged components, ensuring the integrity of the mitochondrial network and limiting the production of harmful reactive oxygen species (ROS) that damage DNA, lipids, and proteins, leading to cell death.

As mitochondria accrue damage, specific mitoQC pathways are activated, where pathway selectivity is influenced by the level of impairment and by the particular mitochondrial protein or lipid cargo that is damaged [2]. Whole mitochondrial clearance, typical following complete depolarisation of the inner mitochondrial membrane (IMM), is predominantly mediated by the autophagy pathway. Here, irreversibly damaged whole mitochondria are encapsulated within an autophagosome and subsequently trafficked to the lysosome for degradation. One of the most well studied pathways for whole mitochondrial clearance is the ubiquitin‐dependent mechanism mediated by the serine/threonine kinase PINK1 and the E3 ubiquitin ligase Parkin [3].

Both of their associated genes, PARK2 and PARK6, are mutated in familial forms of Parkinson's disease, and their loss of function leads to elevated mitochondrial dysfunction [4, 5, 6, 7]. PINK1 acts as a damage sensor to trigger the recruitment and full activation of Parkin, which tags outer mitochondrial membrane (OMM) proteins with polyubiquitin that are recognized by autophagy receptors, optineurin, NDP52, and tax1bp1, recruiting the growing autophagosomal membrane [8]. There do exist alternative ubiquitin‐independent pathways of whole mitochondrial clearance [2], suggesting PINK1 and Parkin may be dispensable. In addition, rather than whole mitochondrial clearance, alternative repair pathways that turnover small regions of the mitochondria also exist and may be favored in response to local oxidative stress or damage. One such pathway, which requires the budding of small 70–100 nm sized vesicles in a Miro1/2‐ and Drp1‐dependent manner, has been shown to also require the activity of PINK1 and Parkin [9, 10, 11]. This mitochondrial‐derived vesicle (MDV) pathway comprises multiple distinct subtypes, which are defined by their derivation, outer membrane only or inner‐membrane/matrix derived, their cargo composition, as well as their destination, to the peroxisome or lysosome [12]. Beyond their response to damage, these MDVs promote the turnover of whole mitochondrial protein complexes at the outer membrane, suggesting a necessary steady state function [10]. Furthermore, recent evidence suggests the MDV pathway may compensate for the loss of whole mitochondrial turnover by LC3‐mediated mitophagy to potentially maintain a healthy mitochondrial network when autophagy may be inactivated [13].

Although it is apparent PINK1 and Parkin play an active role in the MDV pathway, there exist data that conflict with the notion that PINK1 and Parkin are essential for MDV biogenesis [9, 14]. Instead, there is the suggestion that PINK1 may act to repress the formation of MDVs [15] and our own data suggest loss of Parkin leads to an accumulation of MDVs, rather than an inhibition of biogenesis [11]. Therefore, there may be cell type‐specific responses and the driving mechanisms may be influenced by varying physiological levels of essential regulators across the disparate mitoQC pathways. In addition, MDVs may play a role beyond organelle quality control as indicated by their function in mitochondrial antigen presentation influencing the immune response [15]. Thus, the MDV pathway is a complex mechanism that utilizes a range of mitochondrial‐associated and endosomal adaptors that have a promiscuous function across multiple mitoQC pathways, where many cargo types exist and differing routes may be taken. This suggests further research is required to understand the key requirements for MDV formation and entry into the endolysosomal pathway.

Our previous study identified that loss of Parkin results in a selective accumulation of TOM20‐positive MDVs as a result of a defect in their trafficking to a LAMP1‐positive compartment [11]. Therefore herein, we aimed to define the contribution of PINK1 to the MDV pathway and how perturbation of PINK1 in comparison to Parkin influences overall mitochondrial dynamics and quality control. Our results indicate that PINK1 and Parkin loss of function have differing impacts on the MDV pathway, while both are dispensable for the steady state maintenance of the mitochondrial network and for damage‐induced whole mitochondrial clearance. These data collectively indicate that the perturbation of particular mitoQC machinery may compromise unique aspects of MDV biogenesis and trafficking, dependent on cargo identity and destination.

## 2 Materials and Methods

### 2.1 Antibodies and Reagents

Antimycin A (A8674‐25MG) and oligomycin (495455‐10MG) were purchased from Merck, and CCCP (11357056) and Bafilomycin A1 (10295441) were purchased from ThermoFisher. Antibodies used in this study were: LAMP1 (BD Bioscience, 555798, RRID:AB_396132), HA (BioLegend, 901501, RRID:AB_2565006), HA (Cell Signaling Technology, 3724S, RRID:AB_1549585), Parkin (Cell Signaling Technology, 4211, RRID:AB_2159920), PINK1 (Cell Signaling Technology, 6946, RRID:AB_11179069), Mitofusin‐2 (Cell Signaling Technology, 9482, RRID:AB_2716838), COX‐II (MTCO‐2, Abcam, ab110258, RRID:AB_10887758), cytochrome C (BioLegend, 612302, RRID:AB_315775), actin (Proteintech, 66009‐1‐Ig, RRID:AB_2687938), GAPDH (Proteintech, 10494‐1‐AP, RRID:AB_2263076), phospho‐serine 65 ubiquitin (Millipore, ABS1513‐I, RRID:AB_2858191), Atg5 (Cell Signaling Technology, 2630S, RRID:AB_2062340), TOM20 (Abcam; ab78547, RRID:AB_2043078), TOM20 (Santa Cruz, SC‐11415, RRID:AB_2207533), LC3 (MBL, M152‐3, RRID:AB_1279144), LC3 (Novus Biologicals, NB100‐2220, RRID:AB_10003146), PDH E2/E3bp (Abcam, ab110333, RRID:AB_10862029), and PDHA1 (Abcam, ab110330, RRID:AB_10858459).

### 2.2 Cell Culture

SH‐SY5Y cells were maintained in DMEM/Ham's F12 media (ThermoFisher, 11520396) supplemented with 10% FBS (Merck, F0679), 1% non‐essential amino acids (ThermoFisher, 12084947), and 1% penicillin/streptomycin (ThermoFisher). HEK293T and ARPE‐19 cells were cultured in DMEM (Merck, D6429) supplemented with 10% FBS and 1% penicillin/streptomycin. All cells were maintained at 37°C, 5% CO_2_. To induce mitochondrial damage, cells were treated with the combination of 5 μM antimycin A and 10 μM oligomycin, or 20 μM CCCP. Bafilomycin A1 was used at 100 nM.

### 2.3 Plasmid Constructs and Cell Transfection

The HA‐Parkin cassette was subcloned from pRK5‐HA‐Parkin (gift from Ted Dawson; Addgene, RRID:Addgene_17613) into pIRESpuro2, and site‐directed mutagenesis was performed to create the R42P, T240R, R275W, and C431S mutants as previously described [11]. Cells were transfected using Fugene 6 (Promega; E2693) at a 3:1 ratio of transfection reagent volume to μg DNA. Cells were incubated 24 h prior to any experimental processing.

### 2.4 Lentiviral CRISPR‐Cas9 Knockout Generation

The guide RNA targets used were as follows: Exon 2 of Parkin (GGTGGTTGCTAAGCGACAGG), Exon 1 of PINK1 (ATGGCGGTGCGACAGGCGCT), Exon 2 of Atg5 (AAGATGTGCTTCGAGATGTG). These guides were engineered to clone into pLentiCRISPRv1 (Addgene, gift from Feng Zhang) using the BsmBI restriction site with oligonucleotides purchased from Merck. The presence of guide RNA was verified by sequencing with a U6 forward primer (ACTATCATATGCTTACCGTAAC). As a control, wild‐type cells represent an empty vector Lentiviral CRISPR‐Cas9 targeted cell line lacking a gRNA. Lentiviral particles were packaged in HEK293T cells following the transfection of the pLentiCRISPR plasmid alongside psPAX2 (Addgene, RRID:Addgene_12260; gift from Didier Trono) and pMD2.G (Addgene, RRID:Addgene_12259; gift from Didier Trono) using the PEI transfection reagent. Viral supernatant was harvested 48 h post‐transfection and used to infect SH‐SY5Y cells in a 6‐well dish in the presence of 5 μg/mL polybrene at 37°C. Following a 24‐h incubation, media was replaced with growth media containing 1.5 μg/mL puromycin to select Cas9 expressing cells. The level of target protein loss was confirmed by Western blot analysis.

### 2.5 ATP Assay

Cells were plated in replicate 96‐well tissue culture plates at a density of 4 × 10^5^ cells per well with each treatment in duplicate. Following 24‐h incubation, cells were washed twice and replenished with 100 μL DMEM without glucose (ThermoFisher, 11966025) supplemented with 10 mM galactose, 1% nonessential amino acids, 10% dialysed FBS (Sigma Aldrich, F0392), and 1% penicillin/streptomycin or with glucose‐containing growth media, in the absence or presence of 10 μM Oligomycin. Cells were incubated at 37°C for 2 h prior to using plates for either measuring ATP production using the Viral ToxGlo Assay (Promega, G8941) or protein content using a BCA assay. To measure ATP content, media was removed from one plate and 100 μL ATP detection reagent was added to each well, including wells containing media only. Samples were transferred to a white 96‐well plate and luminescence was measured on a Promega GloMax detection system. A background subtraction was performed against media‐only values, and luminescence data was normalized against the total protein content, as measured by the BCA assay, to obtain the relative light units (RLU)/μg of protein. Results represent technical replicate readings across three independent biological replicates for each condition.

### 2.6 Immunofluorescence Microscopy

Cells were plated on either 22 × 22 mm square or 16 mm round glass coverslips in either 6‐well or 12‐well tissue culture treated dishes prior to processing for immunofluorescence microscopy. Cells were fixed in 4% formaldehyde (Polysciences, 04018) in PBS for 20 min at room temperature. Following washes in PBS, cells were permeabilised in 0.02% Triton X‐100 in PBS for 2 min at room temperature. This was followed by washing in PBS for 10 min. Coverslips were blocked in 1% BSA (ThermoFisher; 11403164) in PBS for 20 min at room temperature. Following this, coverslips were incubated with appropriate primary antibodies diluted in PBS, 1% BSA for 1.5 h at room temperature. Coverslips were then washed for 10 min, prior to incubation with secondary antibodies AlexaFluor 488 or 568 goat anti‐rabbit or mouse (ThermoFisher; 10696113, 10032302, 10348072, 10729174) for 45 min at room temperature. Hoechst was purchased from Thermofisher and diluted 1:10,000 in secondary antibody preparation. Coverslips were then washed in PBS, rinsed in dH_2_O, and mounted using Fluorsave (VWR; 345789‐20) on glass slides. Slides were imaged on an Olympus IX83 inverted fluorescence microscope using a 63× or 100× objective, and images were processed and analyzed using Fiji [16].

### 2.7 Western Blot

Cell lysates were collected in either lysis buffer (1% Triton X‐100, 0.1% SDS, 150 mM NaCl, 10 mM Tris–HCl pH 7.4, 5 mM EDTA) supplemented with a protease inhibitor tablet (Merck; 04693159001) or directly harvested in 1× SDS‐Sample buffer (62.5 mM Tris–HCl pH 6.8, 2% SDS, 2.5% β‐mercaptoethanol, 10% Glycerol, 0.005% Bromophenol blue) as whole cell lysates. Cell extracts harvested in lysis buffer were spun at 13,300 RPM at 4°C for 15 min, followed by the collection of supernatant. Cell supernatant was subjected to a BCA protein assay (ThermoFisher; 10678484) and 25 μg of protein was loaded onto SDS‐PAGE. Cell extracts were run on 10%–15% SDS‐PAGE alongside either the EZRun prestained protein standard (ThermoFisher; 10,638,393) or PageRuler prestained protein standard (ThermoFisher; 11812124), prior to transfer to Immobilon‐FL PVDF (ThermoFisher; 10452792). Membranes were blocked in either 5% milk or 3% BSA in TBS, prior to incubation with primary antibody in blocking buffer overnight at 4°C. Membranes were washed in TBS, 0.1% Tween‐20, followed by incubation with IRDye 800CW Goat anti‐mouse or rabbit IgG, or IRDye 680RD Goat anti‐rabbit or mouse IgG at 1:5000 in TBS, 0.1% Tween‐20, 0.04% SDS for 1 h at room temperature. Immunoblotted membranes were imaged with a LiCOR Odyssey and processed using Fiji [16]. Western blots were quantified by gathering the integrated density value in Fiji of a respective immunoprobed band following normalization to either the actin or GAPDH loading controls. Values are presented as either the fold or percentage change from a control or untreated group. In the case of mitofusin‐2, values are presented as the percentage of high molecular weight species against the value of total protein. Graphs were generated in GraphPad Prism and data was analyzed by one‐way ANOVA followed by Tukey's or Dunnett's post hoc analysis for multiple comparisons.

### 2.8 Quantitative Image Analysis

#### 2.8.1 LC3 Colocalisation

Fiji was used to quantify LC3 colocalisation with mitochondria. Background was subtracted from the individual monochrome images of TOM20 and LC3, then each channel was transformed to binary. For the LC3 labeling, the images were thresholded manually for each field of view to highlight LC3 puncta. Pearson's coefficient was applied to assess the proportion of TOM20 colocalising with LC3. Two to four fields of view were analyzed for each independent biological replicate and a two‐way ANOVA with Tukey's post hoc analysis was applied.

#### 2.8.2 MDV Quantitation

Fiji was used to quantify the number of TOM20+ve/PDH−ve and PDH+ve/TOM20−ve MDVs. For each mitochondrial marker, background was subtracted from individual greyscale channels and levels adjusted to maximize signal to noise. The single channel, monochrome images were then merged to create a composite RGB image. To identify and quantify MDVs, one monochrome mitochondrial channel was selected and a single cell was chosen to analyze. Vesicles were labeled using the “point tool” to create a region of interest (ROI). The ROI was added to the ROI manager and then overlaid on the monochrome image from the other mitochondrial marker. Vesicles were then determined to be MDVs based upon the absence of the second mitochondrial marker and the total number of the respective subset of MDVs counted per cell. This analysis was repeated for the alternate MDV subtype. Analysis of at least 7 cells per experimental group was performed across 3 independent biological repeats. A one‐way ANOVA with Tukey's post hoc analysis was applied for statistical tests.

To determine LAMP1 colocalisation with MDVs, first the background was subtracted from individual grayscale images of LAMP1, and levels adjusted to maximize the signal to noise. Mitochondrial markers were processed as previously described above. The number of MDVs positive for LAMP1 was then calculated as a percentage of the total number of MDVs for each individual cell. At least 5 cells per experimental group were included across three independent biological repeats. A one‐way ANOVA with Tukey's post hoc analysis was applied for statistical tests.

#### 2.8.3 Mitochondrial Network Analysis

For the analysis of mitochondrial morphology and connectivity, microscopic images were put through a workflow as previously described using Fiji software [17]. Images labeled for TOM20 were thresholded by applying the following functions “Subtract Background,” “Sigma Filter Plus,” “Enhance Local Contrast,” “Gamma,” “Adaptive threshold,” “Despeckle” and “Remove Outliers”. The resulting binary image was merged with Hoechst labeled nuclei to enable selection of individual cells using the ROI manager and freehand selection tool. Twenty cells were selected per experimental group and individual cells were isolated for analysis. For each cell, the “Analyse Particles” function was used to capture the morphological information associated with area and aspect ratio. The “Skeletonise 2D/3D” plugin was then applied and the “Analyse Skeleton” function was used to measure connectivity information, in this case the number of branches. For each cell, the mean of each measurement was calculated and for each experimental group, the means of each of the 20 cells were taken for statistical analysis. The data were presented as SuperPlots [18].

## 3 Results

### 3.1 PINK1 and Parkin Are Not Required for Whole Mitochondrial Clearance

**FIGURE 1:**
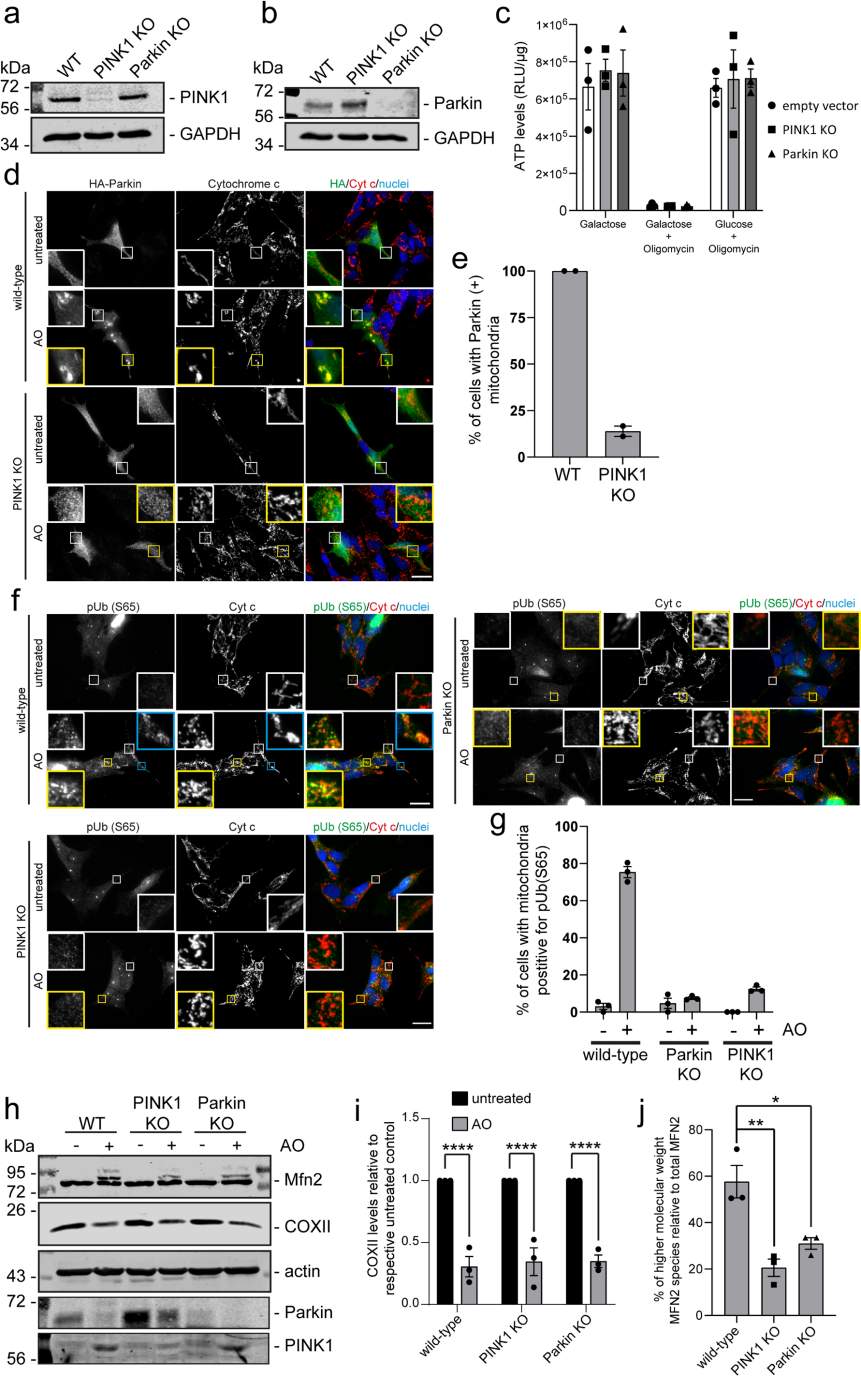
Limited effects of PINK1 and Parkin loss of function on whole mitochondrial clearance. Western blot confirmation of CRISPR‐Cas9 targeted PINK1 (a) and Parkin (b) knockout SH‐SY5Y cells using antibodies to the indicated proteins. Wild‐type (WT) is empty vector Lentiviral CRISPR‐Cas9 targeted cells without a gRNA. (c) ATP assay of empty vector (EV), PINK1 KO, or Parkin KO SH‐SY5Y cells cultured in either Galactose only, Galactose with 10 μM Oligomycin, or glucose with 10 μM Oligomycin for 2 h. Luminescence was measured and normalized to protein content. The data is presented as Relative light units (RLU) per microgram of protein. Each data point represents 1 independent experiment (*n* = 3) and bars represent the average value. (d) Immunofluorescence microscopy of wild‐type (empty vector) or PINK1 KO SH‐SY5Y cells expressing HA‐Parkin, untreated or treated with Antimycin/oligomycin (AO) in combination for 2 h prior to fixation and immunostaining for HA (green) and cytochrome *c* (red). Nuclei labeled with Hoechst (blue). (e) The percentage of cells with Parkin positive (+) mitochondria following AO treatment were quantified and represented as the average of 2 independent experiments (each data point). (f) Immunofluorescence microscopy of wild‐type (empty vector), PINK1 KO or Parkin KO cells untreated or treated with AO for 2 h prior to fixation and immunostaining for phospho‐Serine 65 ubiquitin, S65 (green) and cytochrome c (red). Nuclei are labeled with Hoechst (blue). (g) The percentage of cells with mitochondria positive for pUb (Ser65) were quantified. Each data point represents 1 independent experiment (*n* = 3) and bars represent the average value. (h) Western blot analysis of wild‐type (empty vector), PINK1 KO, or Parkin KO SH‐SY5Y cells untreated or treated with AO for 24 h. Immunoblot analysis was performed using antibodies specific to indicated proteins. (i) Quantitation of CoxII Western blot data. Values for CoxII was normalized to the Actin loading control and data was presented as the fold change in CoxII following AO treatment compared to untreated controls. Each data point represents 1 independent experiment (*n* = 3) and bars represent the average value. (j) The level of the high molecular species of Mitofusin‐2 (MFN2) was quantified and represented as the percentage of the total MFN2. Each data point represents 1 independent experiment (*n* = 3) and the bars represent the average value. Error bars are the SEM. Scale bar = 20 μm. *p < 0.05, **p < 0.01, ****p < 0.0001.

The dominant ubiquitin‐dependent method of mitochondrial degradation is mediated by the serine/threonine kinase PINK1 and the E3 ubiquitin ligase Parkin. Much of the previous literature has relied on the forced overexpression of Parkin to delineate mechanisms of the pathway. However, this offsets the balance between disparate mitochondrial quality control (mitoQC) pathways and therefore does not provide insight into the physiological dependence of PINK1 and Parkin on differential mitoQC pathways. In order to understand their requirement across different mitoQC pathways, we first developed loss of function models using the SH‐SY5Y cell line which express high physiological levels of both PINK1 and Parkin. We used CRISPR‐Cas9 to generate both PINK1 and Parkin knockout (KO) SH‐SY5Y models and confirmed complete loss of protein expression by Western blot (Figure 1a,b). Importantly, neither loss of PINK1 nor Parkin led to any significant changes in the cells ability to produce ATP in the presence of galactose or total mitochondrial load in cells grown in either glucose or galactose (Figure 1c and Figure S1a,c). In addition, there was no impact on the steady state mitochondrial network organization and morphology, or its response to Antimycin–Oligomycin (AO) induced damage (Figure S1a,b). However, PINK1 loss led to an elevation in Parkin expression and a decrease in Parkin translocation to mitochondria in response to AO‐induced damage (Figure 1d,e,h). In contrast, loss of Parkin did not influence PINK1 accumulation in response to AO‐induced mitochondrial damage (Figure 1h).

**FIGURE 2:**
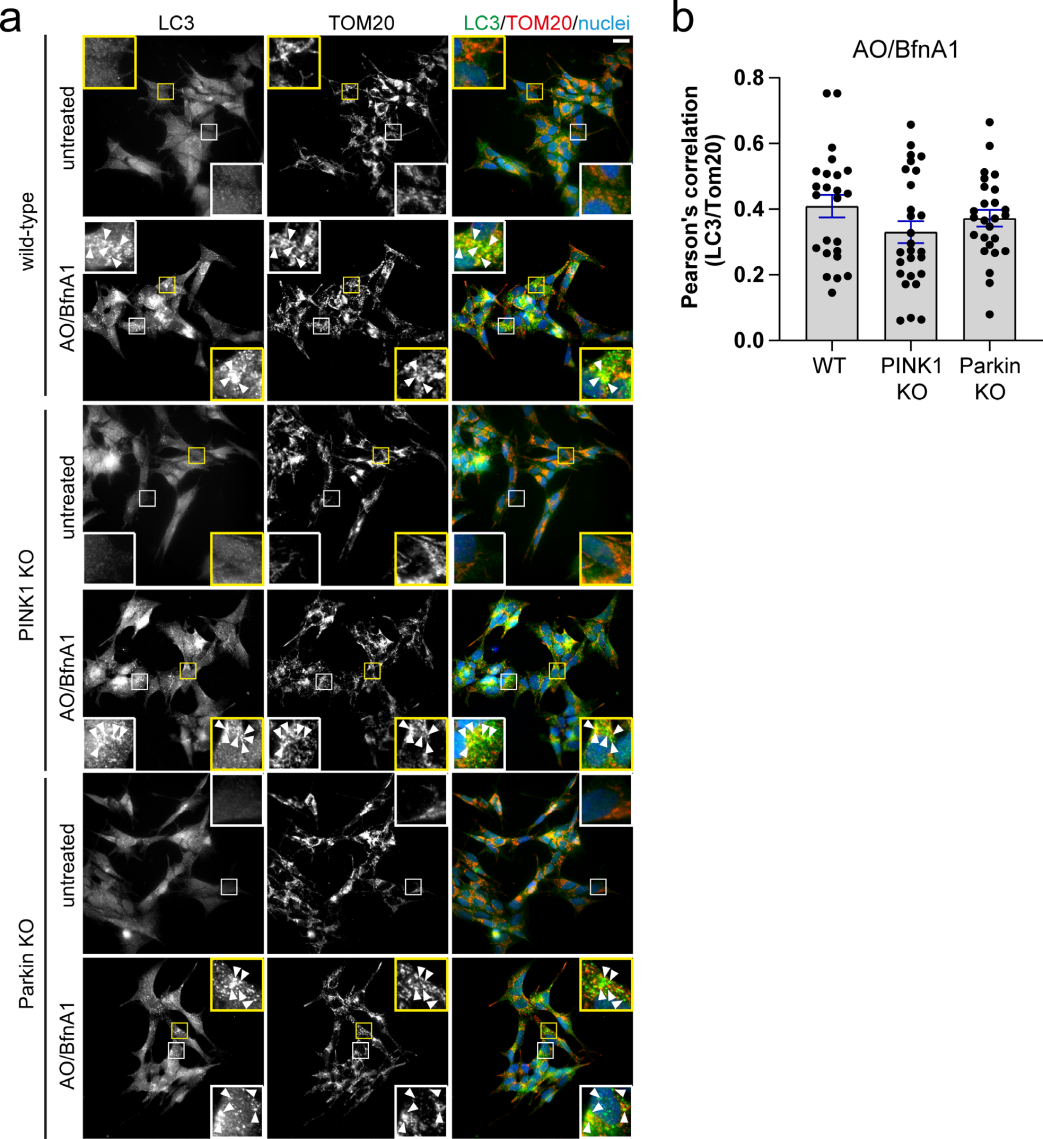
Loss of PINK1 or Parkin has no effect on LC3 recruitment to damage‐induced mitochondria. (a) Immunofluorescence microscopy of SH‐SY5Y WT (EV), PINK1 KO, or Parkin KO cells either left untreated or treated with AO in combination with 100 nM Bafilomycin A1 for 6 h. Cells were immunostained for LC3 (green), TOM20 (red), and nuclei are labeled with Hoechst (blue). Arrowheads indicate areas of LC3 and TOM20 colocalisation. Scale bar = 20 μm. (b) Quantitative analysis of LC3 and TOM20 colocalisation from immunofluorescent images represented by the Pearson's correlation coefficient. Each data point represents 1 cell, gathered from three independent biological replicates (> 8 cells/experiment). Bar is the average value and error bars represent the SEM.

We then aimed to assess the impact of the loss of PINK1 and Parkin expression on mitophagy and whole mitochondrial clearance. One of the first indicators of a PINK1‐Parkin dependent process is the accumulation of phosphorylated ubiquitin at Serine 65 (pUb) [19]. We found that the loss of either PINK1 or Parkin had a significant effect on the ability of cells to mark mitochondria with pUb in response to AO‐induced damage (Figure 1f,g). In addition, following Parkin recruitment to mitochondria, the outer membrane protein Mitofusin‐2 (Mfn2) is ubiquitylated and degraded by the proteasome [20]. Our data indicate that the loss of both PINK1 and Parkin decreases the accumulation of the high molecular weight, ubiquitylated species of Mfn2 following AO‐induced damage (Figure 1h,j). Although the initial steps of the PINK1‐Parkin dependent pathway appear to be inactive, we did not observe any defect in the ability of cells to clear whole mitochondria from the cytoplasm, as illustrated by the efficient degradation of COX‐II following 24‐h treatment with AO (Figure 1h–j). We corroborated our results in APRE‐19 cells, which have detectable levels of PINK1 and Parkin, although at varying levels compared to SH‐SY5Y and HEK293T cells (Figure S2a). In ARPE‐19 cells, the loss of PINK1 had no significant impact on whole mitochondrial turnover (Figure S2b,c), despite evidence of pUb present around mitochondria in wild‐type cells following AO‐induced damage (Figure S2d). Providing further evidence for an alternative, compensatory PINK1/Parkin‐independent mitophagic pathway, we observe the accumulation of LC3 on mitochondria following AO‐induced mitochondrial damage in the presence of the vATPase inhibitor Bafilomycin A1 (BfnA1) in our PINK1 and Parkin KO SH‐SY5Y cells (Figure 2). The use of Bafilomycin A1 allowed us to assess the extent of LC3 accumulation on mitochondria without being influenced by the turnover rate. The ability of LC3 to localize to mitochondria in response to AO‐induced damage was corroborated in ARPE‐19 cells lacking PINK1 (Figure S2e). These data collectively suggest that despite the complete loss of PINK1 and Parkin, SH‐SY5Y cells can efficiently clear damaged whole mitochondria via alternative, compensatory autophagic mechanisms.

### 3.2 Disruption of Autophagy via Atg5 Loss of Function Does Not Impact Whole Mitochondrial Turnover, Although It Leads to an Elevation in PDH‐Positive MDVs

**FIGURE 3:**
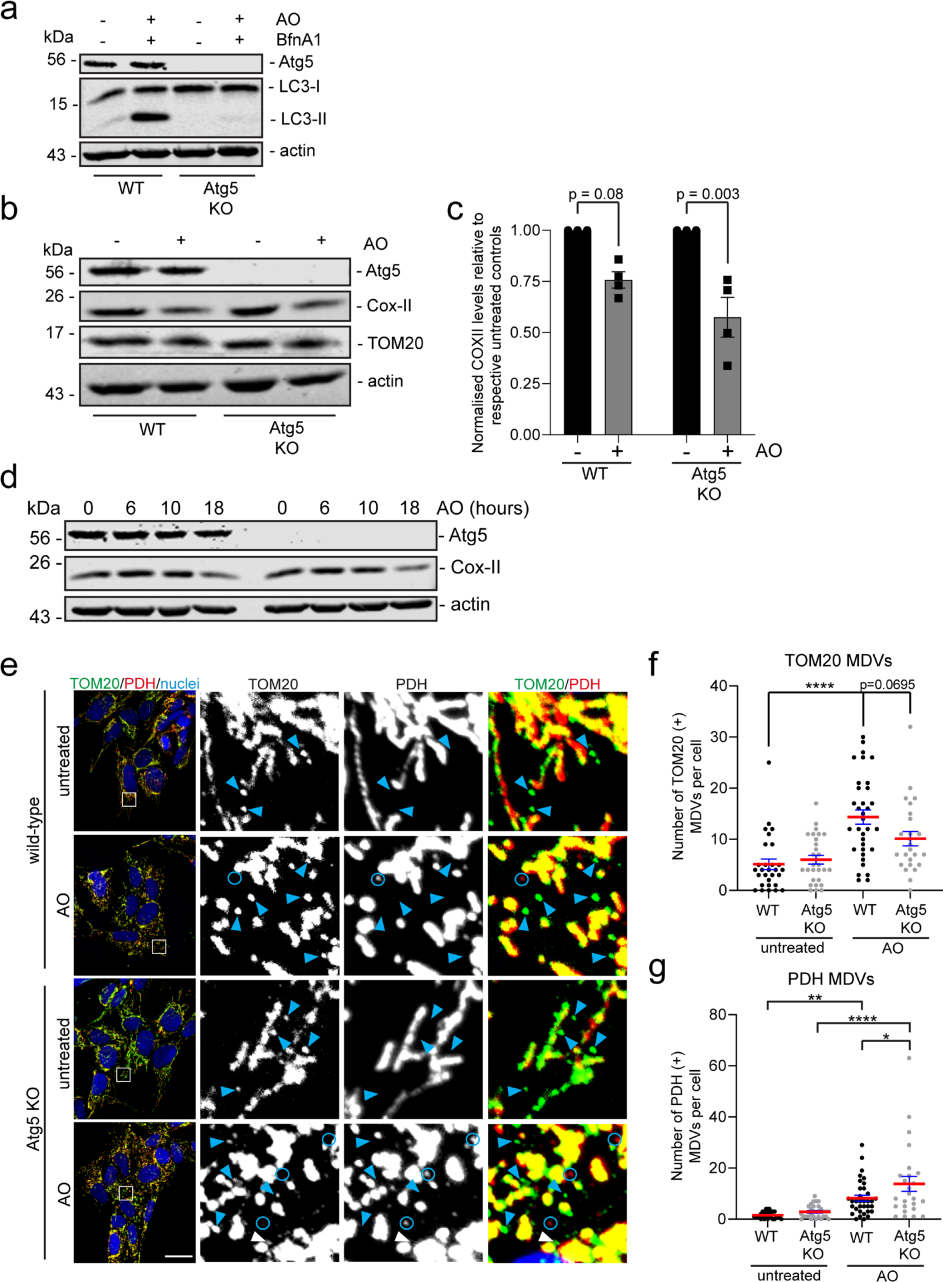
Absence of Atg5 expression leads to a selective elevation of PDH (+) MDVs, but no effect on bulk mitochondrial clearance following induced damage. (a) Western blot analysis of cell lysates harvested from SH‐SY5Y wild‐type (WT) or Atg5 KO cells either untreated or treated with 100 nM Bafilomycin A1 (BfnA1) for 2 h. Membranes were probed with antibodies against indicated proteins. (b) Western blot analysis of cell lysates harvested from SH‐SY5Y wild‐type (WT) or Atg5 KO cells either untreated or treated with AO for 24 h. Membranes were probed with antibodies against indicated proteins. (c) Quantitation of Cox‐II levels from Western blot data in (b) normalized to actin loading control, presented as fold change from untreated controls. (d) Western blot analysis of cell lysates harvested from SH‐SY5Y wild‐type (WT) or Atg5 KO cells either untreated or treated with AO for 6, 10, or 18 h. Membranes were probed with antibodies against indicated proteins. (e) SH‐SY5Y WT (EV) and Atg5 KO cells either left untreated or treated with AO for 2 h prior to processing for immunofluorescence microscopy. Cells were immunostained for TOM20 (green), PDH E2/E3 bp (red), and nuclei labeled with Hoechst (blue). Lower magnification images (Scale bar = 20 μm) are in left column and expanded higher magnification insets are on the right. Blue arrowheads indicate TOM20 (+) MDVs (TOM20‐positive/PDH‐negative) and blue circles indicate PDH (+) MDVs (PDH‐positive/TOM20‐negative). Quantitative analysis of TOM20 (f) or PDH (g) positive MDVs from SH‐SY5Y WT or Atg5 KO cell immunofluorescent images. Each data point represents 1 cell gathered from 3 independent experiments. Red bar represents the mean and error bars are the SEM. *p < 0.05, **p < 0.01, ****p < 0.0001.

We next aimed to determine whether targeting Atg5 to block autophagy would impact whole mitochondrial turnover or repair pathways such as MDVs. We used CRISPR‐Cas9 to develop Atg5 knockout SH‐SY5Y cells, which exhibited a complete loss of Atg5 protein expression and a substantial decrease in LC3 lipidation, as illustrated by a lack of LC3‐II in the presence of BfnA1, confirming loss of canonical autophagosome formation (Figure 3a). However, although there is a complete loss of LC3 lipidation in Atg5 KO cells, they are still capable of clearing whole mitochondria following AO‐induced damage, as illustrated by the degradation of COX‐II to a level comparable to wild‐type cells (Figure 3b,c). In addition, across a time course of AO‐induced damage, there are no defects in the rate of COX‐II loss (Figure 3d). Recent evidence suggests that the MDV pathway is a repair mechanism that may compensate for the absence of a LC3‐mediated mitophagic pathway [13]. We therefore quantified both the number of TOM20 (+) (TOM20‐positive/PDH‐negative) and PDH (+) (PDH‐positive/TOM20‐negative) MDVs in SH‐SY5Y Atg5 KO cells. These data suggest no significant difference in the number of TOM20 (+) MDVs in Atg5 KO cells despite an AO‐induced damage response (Figure 3e,f), which is consistent with Soubannier et al. [21], but contrasts with Towers et al. [13]. We also assessed the number of PDH (+) MDVs in SH‐SY5Y Atg5 KO cells. These data identify a significant increase in this subclass of MDVs in response to AO‐induced damage following loss of Atg5 expression (Figure 3e,g), consistent with previous literature [13]. These data illustrate that despite no impact on whole mitochondrial turnover in the absence of Atg5‐mediated autophagy, there are disparate effects on MDV flux between different subclasses of MDVs, suggesting cargo‐dependent impacts.

### 3.3 Loss of Parkin Leads to an Accumulation of TOM20‐Positive MDVs, Which Can Be Rescued by Reintroduction of Wild‐Type Parkin, but Not Pathogenic Mutants

**FIGURE 4:**
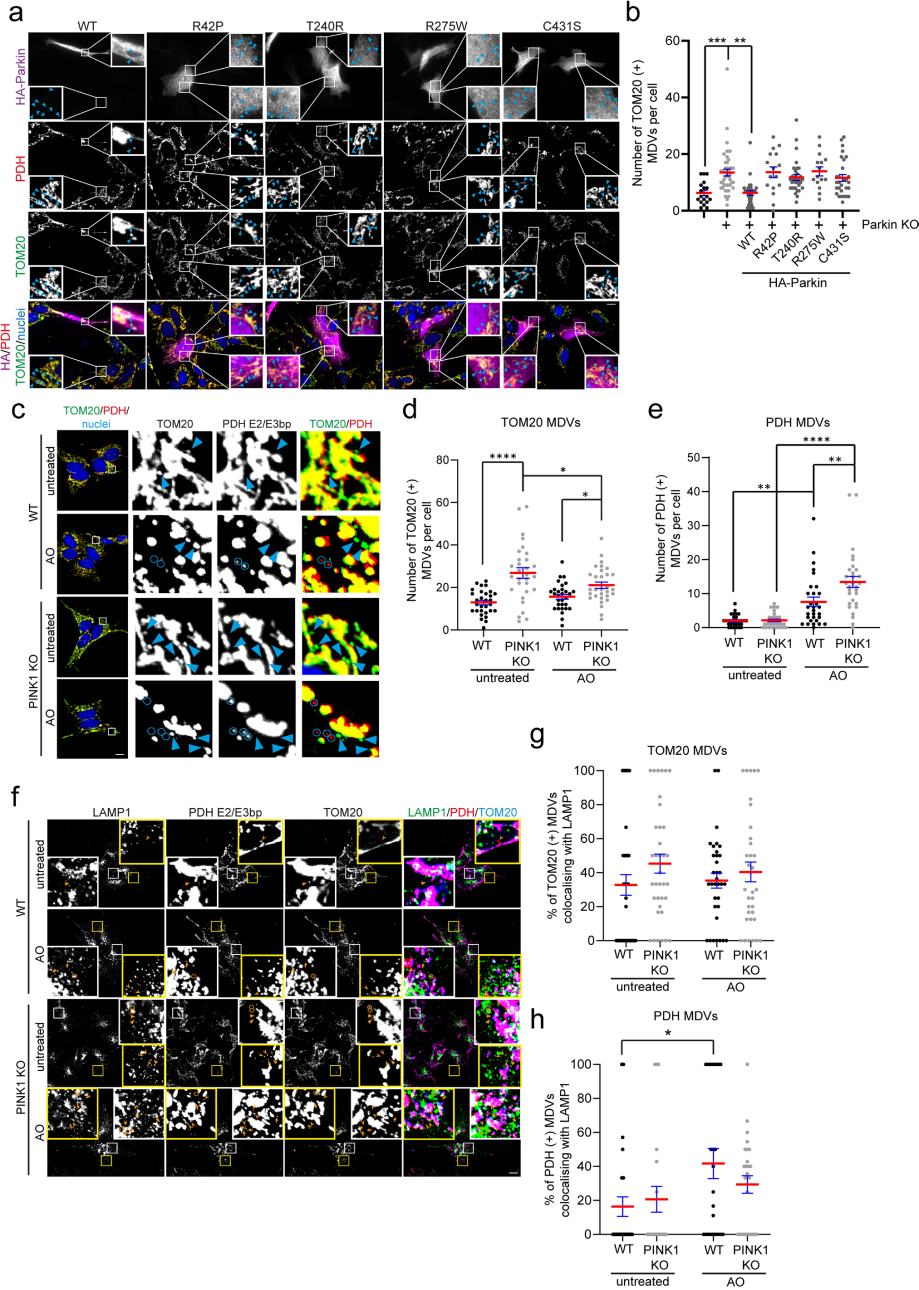
Loss of PINK1 results in an accumulation of MDVs, not due to a defect in MDV trafficking to a LAMP1 compartment. (a) SH‐SY5Y Parkin KO cells expressing either wild‐type (WT), R42P, T240R, R275W, or C431S HA‐Parkin were treated with AO for 2 h prior to processing for immunofluorescence microscopy. Cells were immunostained for HA (magenta), TOM20 (green), PDH E2/E3bp (red), and nuclei were labeled with Hoechst (blue). Blue arrowheads indicate TOM20 (+) MDVs. Scale bar = 10 μm. (b) Quantitation of TOM20 (+) MDVs from immunofluorescence images of WT (EV) or Parkin KO cells expressing WT or mutants of HA‐Parkin. Each data point represents the number of TOM20 (+) MDVs in an individual cell, from 3 independent experiments (> 5 cells/experiment). Red bar represents the mean and error bars represent the SEM. (c) SH‐SY5Y WT (EV) and PINK1 KO cells either left untreated or treated with AO for 2 h prior to processing for immunofluorescence microscopy. Cells were immunostained for TOM20 (green), PDH E2/E3 bp (red), and nuclei labeled with Hoechst (blue). Lower magnification images (Scale bar = 10 μm) are in left column and expanded higher magnification insets are on the right. Blue arrowheads indicate TOM20 (+) MDVs and blue circles indicate PDH (+) MDVs. Quantitative analysis of TOM20 (d) or PDH (e) positive MDVs from SH‐SY5Y WT or PINK1 KO immunofluorescent images. Each data point represents 1 cell gathered from three independent experiments. Red bar represents the mean and error bars are the SEM. (f) SH‐SY5Y WT (EV) and PINK1 KO cells either left untreated or treated with AO for 2 h prior to processing for immunofluorescence microscopy. Cells were immunostained for LAMP1 (green), PDH E2/E3 bp (red), and TOM20 (blue). Orange arrowheads indicate TOM20 (+), LAMP1 (−) MDVs; orange circles indicate TOM20 (+), LAMP1(+) MDVs; orange indented arrowheads indicate PDH (+), LAMP1 (−) MDVs; and orange arrows indicate PDH (+), LAMP1(+) MDVs. Scale bar = 10 μm. Percentage of (g) TOM20 (+) or (h) PDH (+) MDVs colocalising with LAMP1 from SH‐SY5Y WT or PINK1 KO immunofluorescent images. Each data point represents 1 cell gathered from three independent experiments. Red bar represents the mean and error bars are the SEM. *p < 0.05, **p < 0.01, ***p < 0.001, ****p < 0.0001.

Our previous data from SH‐SY5Y cells suggests that loss of Parkin results in an accumulation of TOM20 (+) MDVs, due to an inability to efficiently traffic to a LAMP1‐positive compartment [11]. This contrasts with inner‐membrane derived MDVs, where previous work has indicated loss of Parkin does not result in an accumulation of PDH (+) MDVs, but rather an inhibition in their formation [11, 22]. In this study we aimed to determine whether the impact of Parkin loss of function on TOM20 (+) MDVs can be rescued by re‐expression of HA‐tagged WT Parkin or associated pathogenic mutants. We assessed the number of TOM20 (+) MDVs in SH‐SY5Y Parkin KO cells expressing wild‐type HA‐Parkin (WT) or the pathogenic mutants R42P, T240R, R275W, and C431S HA‐Parkin. Our data indicate that while the reintroduction of WT Parkin can rescue the defect in MDV accumulation, these pathogenic mutants are unable to rescue this phenotype (Figure 4a,b). Our data collectively indicate that Parkin is required for MDV trafficking and defects in Parkin localisation or ligase activity are incapable of replacing wild‐type function with respect to the MDV pathway.

### 3.4 Loss of PINK1 Leads to an Accumulation of MDVs, Which Is Not Due to a Defect in Their Trafficking to a LAMP1‐Positive Compartment

It has been suggested by previous studies that loss of PINK1 inhibits the formation of inner‐membrane, PDH (+) MDVs [9, 14]. However, little is known about the requirement for PINK1 on outer‐membrane, TOM20 (+) MDV formation or trafficking. We therefore used CRISPR‐Cas9 generated SH‐SY5Y PINK1 KO cells to assess the levels of both TOM20 (+) and PDH (+) MDVs. Our data indicate that loss of PINK1 leads to an increase in TOM20 (+) MDVs both at steady state and following AO‐induced mitochondrial damage (Figure 4c,d), which is partially corroborated in a PINK1 KO ARPE‐19 cell line whereby we identify an elevated level of TOM20 (+) MDVs at steady state (Figure S3). However, loss of PINK1 does not lead to a baseline increase in PDH (+) MDVs, but rather only following AO‐induced mitochondrial damage (Figure 4c,e). Since our previous work indicated that the accumulation of MDVs in response to Parkin loss of function was due to a defect in trafficking to a LAMP1‐positive compartment [11], we next assessed whether PINK1 acted similarly. To assess MDV trafficking to the lysosome, we quantified the extent of colocalisation of TOM20 (+) or PDH (+) MDVs with LAMP1, a lysosome marker. A decrease in MDV colocalisation with LAMP1 compared to controls would indicate a defect in trafficking to the lysosome, whereas an elevation of MDV colocalisation with LAMP1 may indicate increased flux through the pathway. Here, our results indicate that despite PINK1 KO cells having an increased level of TOM20 (+) MDVs (Figure 4c,d), this was not due to a defect in trafficking to a LAMP1‐positive compartment (Figure 4f,g), since there were similar levels of MDVs colocalising with LAMP1 compared to wild‐type controls. In contrast to Parkin KO cells [11], an elevation in PDH (+) MDVs following AO‐induced damage was identified in PINK1 KO cells (Figure 4c,e). However, we identified no impact of PINK1 loss of function on the percentage of MDVs trafficking to a LAMP1‐positive compartment (Figure 4f,h). Collectively, our data indicate that loss of PINK1 leads to an increase in both steady state TOM20 (+) MDVs and damage‐induced PDH (+) MDVs, but this is not due to a defect in their trafficking to the lysosome, instead it is suggestive of increased MDV flux.

## 4 Discussion

PINK1 and Parkin are essential regulators of ubiquitin‐dependent whole mitochondrial clearance by mitophagy. However, it remains to be understood their contribution more broadly to mitoQC and their precise function in locally induced repair routes such as the MDV pathway [2]. In this study, we aimed to clarify the requirement for PINK1 in mitochondrial function and quality control at steady state and in response to stress. Our results indicate that PINK1 is dispensable for whole mitochondrial clearance by mitophagy; however, its loss of function leads to elevated levels of MDVs. Importantly, unlike the case for Parkin loss of function, this accumulation of MDVs in PINK1 KO cells is not due to a defect in trafficking to a LAMP1 positive compartment. Instead, these data are suggestive of an increased MDV flux in cells with dysfunctional PINK1.

We aimed to determine whether loss of PINK1 negatively impacts the steady state function of mitochondria. However, our results indicate no substantial impacts on mitochondrial network morphology or ATP production following loss of PINK1 or its partner in mitophagy Parkin, suggesting limited steady state damage in this context. This is consistent with recent studies which suggest loss of Parkin in mouse models does not cause substantial alterations in mitochondrial function [23]. In the case of PINK1, its loss of function has been indicated to only mildly alter mitochondrial organization and function in young mice or at steady state, however, mitochondrial organization and function is severely potentiated under stress or during aging [24, 25]. Although our data doesn't support a steady state defect, it may be the case that loss of PINK1 leads to a progressive loss of mitochondrial function, whereby stress exacerbates an underlying defect leading to progressive mitochondrial damage. As a result, in a pathway where there is limited redundancy, this may lead to the elevated MDV flux we observe in response to localized mitochondrial damage. It has also been suggested that PINK1 may repress MDV formation [15], which is consistent with our results herein. We additionally observe elevated MDVs in both SH‐SY5Y and ARPE‐19 cells lacking PINK1 expression, suggesting the role of PINK1 is not influenced greatly by the variable and restrictive cellular expression of Parkin.

The mechanisms driving the formation of defined subtypes of MDVs may require distinct machinery due to differences in membrane topology, cargo, and end destination [9, 26]. Earlier studies suggest that PINK1 as well as Parkin are required for the formation of inner‐membrane derived MDVs in a STX17 dependent manner [14, 22]. Although our previous data partially supports these findings as indicated by a suppression of inner‐membrane derived MDVs following Parkin loss of function [11], the data herein suggests the loss of PINK1 appears not to inhibit the formation of inner‐membrane derived MDVs, but rather increase their number following damage in SH‐SY5Y cells. In contrast, the formation of outer‐membrane derived, or TOM20‐positive MDVs, is elevated following both Parkin and PINK1 loss of function, albeit due to different mechanisms, but importantly suggesting no detectable defect in the formation of this particular MDV subtype [11] (Figure 4). Our data also suggests Parkin requires its ligase activity and membrane localisation to elicit its function on MDV trafficking. The differential requirement for machinery driving the formation of inner‐membrane versus outer‐membrane derived MDVs has been noted in the literature [11, 14, 22, 27, 28], which is compounded by variation in these requirements across different cellular models. For example, previous work has shown that inner‐membrane derived MDVs selectively require the sorting nexin SNX9 for their formation [15, 28], however data has been conflicted on the role of SNX9 in the formation of outer‐membrane derived MDVs [10, 13, 28]. Therefore, future work to define the mechanisms governing the formation and trafficking of different subtypes of MDVs is of critical importance to fully understand their function during the mitochondrial stress response.

The mitochondrial quality control system in higher eukaryotes comprises a series of multi‐layered pathways that ensure mitochondrial health and function across a range of stress levels, with many requiring distinct mechanisms [2]. There exists a range of defenses, from enzyme‐mediated anti‐oxidant pathways, degradation of misfolded proteins, to turnover of fully depolarized whole mitochondria [29]. This fail‐safe method of quality control prevents unwanted damage from accruing, limiting cell dysfunction and death. Therefore, where defects may arise, compensation across related pathways occurs to maintain cell homeostasis. There is recent precedent for alterations in one discrete mitoQC pathway having impacts on parallel pathways. This was recently identified following Atg7 loss of function in a breast cancer cell line, which resulted in a compensatory increase in outer‐membrane derived MDV flux [13]. However, where we have deleted Atg5 in this study, SH‐SY5Y cells exhibit no significant change in the number of TOM20‐positive MDVs, while having elevated levels of damage‐induced PDH‐positive MDVs, albeit to a low level. These data suggest there may be varying levels of compensation across the MDV pathway, which likely depends on the cellular context and the type of cargo.

Based on this study and the work of others, it is apparent there is significant redundancy and overlap built into the mitochondrial quality control systems to cope with chronic and accumulating stress, which includes the existence of ubiquitin‐dependent and ubiquitin‐independent mechanisms, as well as lipid‐ and endosomal‐mediated pathways [2]. However, the MDV pathway, which responds to oxidative stress and localized damage, may be more susceptible to dysfunction, whereby the function of essential regulators that work across multiple pathways is not dispensable. Therefore, defining how particular mitochondrial cargoes are packaged into inner‐membrane or outer‐membrane derived MDVs and the machinery required for their routing to destinations such as the lysosome or peroxisome will be of critical importance to understand their significance in maintaining cellular homeostasis and how their dysfunction impacts neurodegenerative pathologies, such as Parkinson's disease.

## Author Contributions

C.L.C. performed a majority of the experiments, analyzed data, and edited manuscript. C.R. performed experiments and analyzed data. N.J.T. devised some of the analysis methods and experimental tools. D.A.T. led and conceived study, performed experiments, and drafted manuscript.

## Conflicts of Interest

The authors declare no conflicts of interest.

## Supporting information


Data S1

## Data Availability

The raw data that support the findings of this study can be made available by the authors upon reasonable request.
