# Supplementary material for: PINK1 Loss of Function Selectively Alters the Mitochondrial‐Derived Vesicle Pathway
Source: FASEB Bioadv. 2025 Jul 10;7(7):e70030. doi: 10.1096/fba.2024-00200 (PMC12242855; doi:10.1096/fba.2024-00200)
Supplement: Supplementary file 1 — Data S1 [file FBA2-7-e70030-s001.pdf]

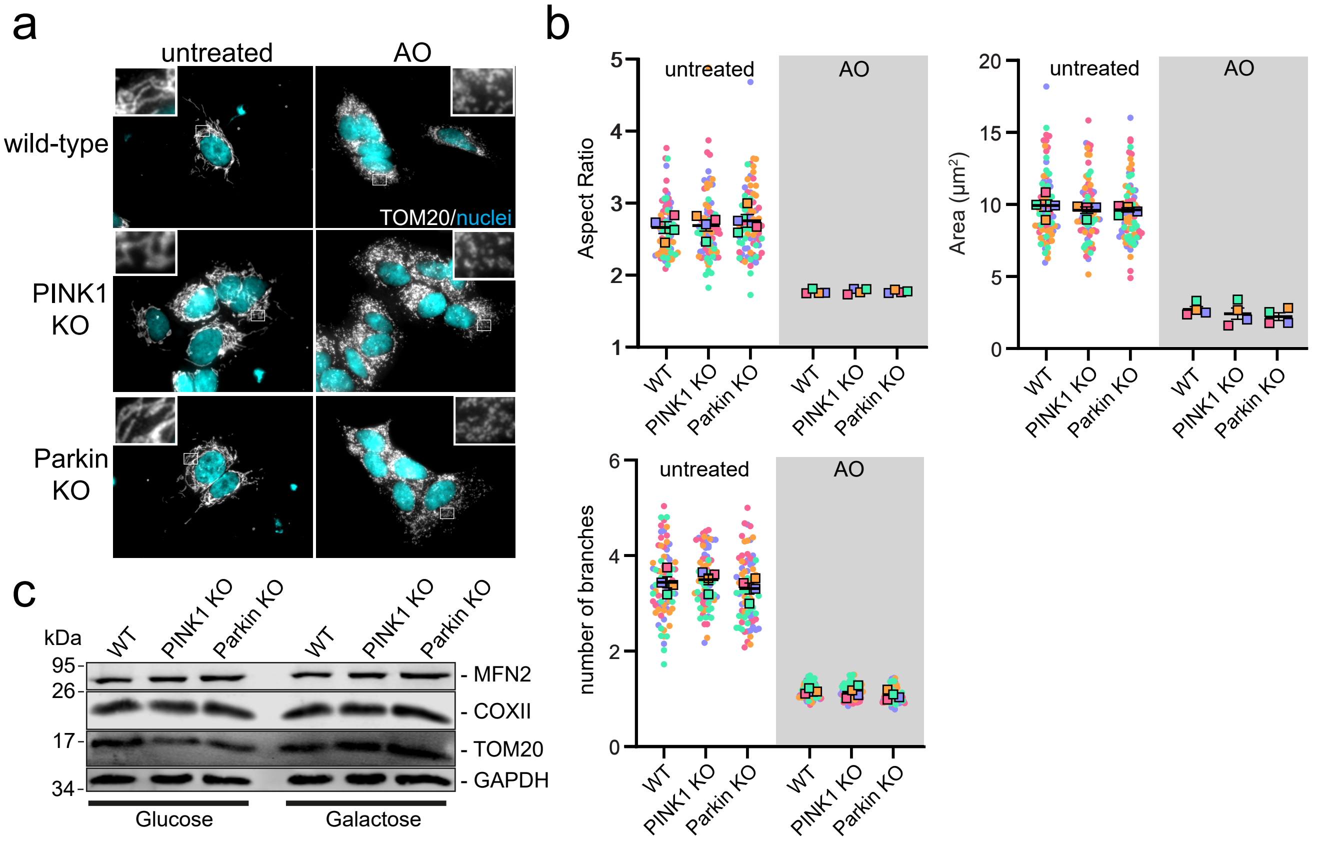

Supplementary Figure 1. No significant defect in mitochondrial load or morphology following PINK1 or Parkin loss of function in SH-SY5Y cells. (a) Mitochondrial network in SH-SY5Y wild-type (WT), PINK1 KO, or Parkin KO cells either untreated or treated with AO for 6 hours. Cells were processed for immunofluorescent imaging followed by immunostaining for TOM20 (grey) and labelling nuclei with Hoechst (Cyan). (b) Quantitative mitochondrial network analysis of TOM20 immunostained images capturing the aspect ratio, area, or number of branches from untreated or AO treated WT, PINK1 KO, or Parkin KO cells. (c) Western blot analysis of lysates harvested from SH-SY5Y WT (EV), PINK1 KO, or Parkin KO cells grown in either glucose-rich or galactose-rich media. Membranes were immuno-probed with antibodies against the indicated proteins.

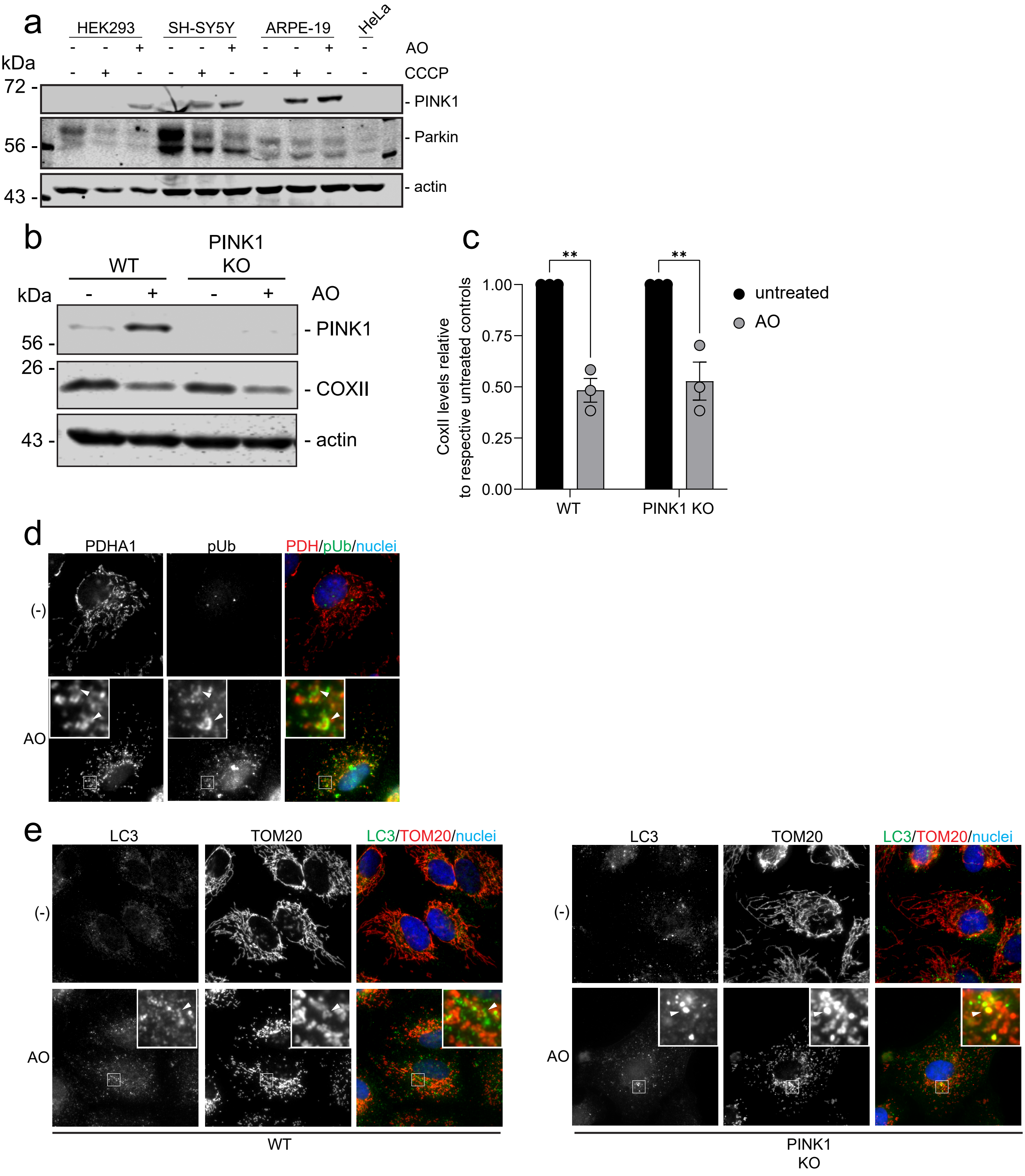

Supplementary Figure 2. ARPE19 PINK1 KO cells show no defect in AO-induced whole mitochondrial turnover. (a) Western blot analysis of lysates harvested from HEK293T, SH-SY5Y, and ARPE-19 cells left untreated or treated with CCCP or AO for 24 hours. Membranes were immunoprobed with antibodies against indicated proteins. (b) ARPE-19 wild-type (WT) and PINK1 KO cells were left untreated or treated with AO for 24 hours prior to harvesting of lysates for Western blot analysis. Membranes were immunoprobed with antibodies against indicated proteins. (c) CoxII intensity levels were normalised to the actin loading control prior to presenting as fold change from the respective cell line untreated control. ** p<0.01 (d) ARPE-19 wild-type cells were left untreated (-) or treated with AO for 6 hours prior to processing for immunofluorescence microscopy. Cells were immunolabelled for PDHA1 (red) and phospho-Serine 65 ubiquitin (pUb; green), while nuclei were labelled with Hoescht (blue). Arrowheads indicate pUb labelled mitochondria. (e) ARPE-19 wild-type (EV control) or PINK1 KO cells were left untreated (-) or treated with AO for 6 hours prior to processing for immunofluorescence microscopy. Cells were immunolabelled for LC3 (green) and TOM20 (red), while nuclei were labelled with Hoescht (blue). Arrowheads indicate areas of LC3 localisation to mitochondria.

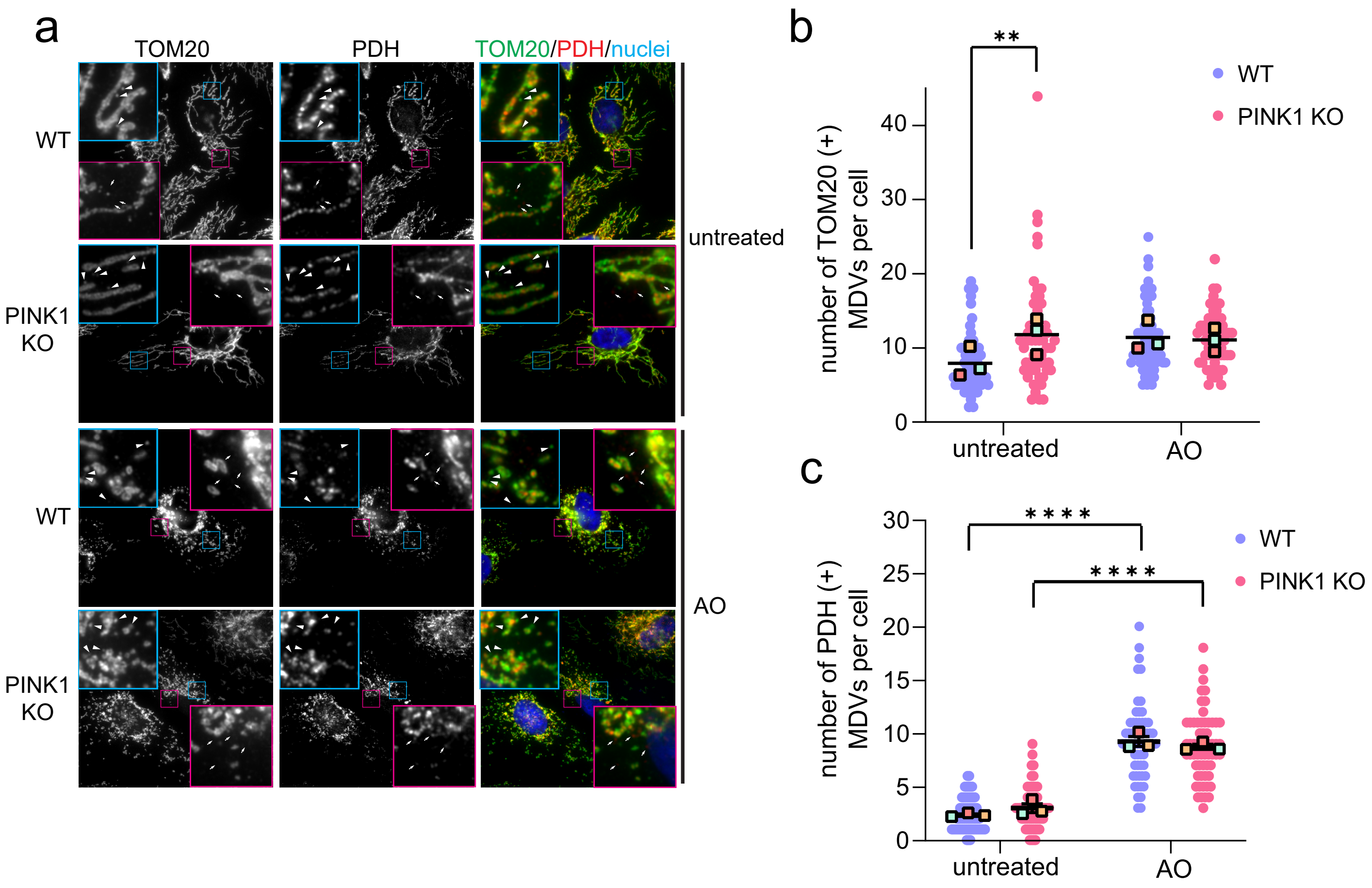

Supplementary Figure 3. ARPE19 PINK1 KO cells display elevated numbers of TOM20-positive MDVs. (a) ARPE-19 wild-type (WT) and PINK1 KO cells were left untreated or treated with AO for 2 hours prior to processing for immunofluorescence microscopy. Wild-type are empty vector Lentiviral CRISPR-Cas9 targeted cells without a gRNA. Cells were immunostained for TOM20 (green) and PDHA1 (red), while the nuclei were labelled with Hoechst (blue). Arrowheads indicate TOM20-positive (+) MDVs within cyan delimited boxes and arrows indicate PDH-positive (+) MDVs within magenta delimited boxes. (b) TOM20 (+) MDVs and (c) PDH (+) MDVs were quantified per cell. Each data point represents a single cell collected from 3 independent biological replicates (60 cells/experimental group), the line represents the overall mean, and each coloured box represents the individual biological replicate mean. ** $p<0.01$, ****$p<0.0001$

Collier et al., Supplementary Figure 3
